# The nasal microbiome in patients suffering from non-steroidal anti-inflammatory drugs-exacerbated respiratory disease in absence of corticosteroids

**DOI:** 10.3389/fimmu.2023.1112345

**Published:** 2023-04-14

**Authors:** Tina J. Bartosik, Nicholas J. Campion, Kilian Freisl, David T. Liu, Katharina Gangl, Victoria Stanek, Aldine Tu, Petra Pjevac, Bela Hausmann, Julia Eckl-Dorna, Sven Schneider

**Affiliations:** ^1^ Department of Otorhinolaryngology, General Hospital and Medical University of Vienna, Vienna, Austria; ^2^ Joint Microbiome Facility of the Medical University of Vienna and the University of Vienna, Vienna, Austria; ^3^ Department of Microbiology and Ecosystem Science, Centre for Microbiology and Environmental Systems Science, University of Vienna, Vienna, Austria; ^4^ Department of Laboratory Medicine, Medical University of Vienna, Vienna, Austria

**Keywords:** microbiome, N-ERD, CRS, CRSwNP, chronic rhinosinusitis, aspirin-exacerbated respiratory disease, 16S rRNA gene amplicon sequencing

## Abstract

Chronic rhinosinusitis (CRS) is a chronic inflammatory disease phenotypically classified by the absence (CRSsNP) or presence of nasal polyps (CRSwNP). The latter may also be associated with asthma and hypersensitivity towards non-steroidal anti-inflammatory drugs (NSAID) as a triad termed NSAID-exacerbated respiratory disease (N-ERD). The role of the microbiome in these different disease entities with regard to the underlying inflammatory process and disease burden is yet not fully understood. To address this question, we measured clinical parameters and collected nasal samples (nasal mucosal fluids, microbiome swabs from middle meatus and anterior naris) of patients suffering from CRSsNP (n=20), CRSwNP (n=20) or N-ERD (n=20) as well as from patients without CRS (=disease controls, n=20). Importantly, all subjects refrained from taking local or systemic corticosteroids or immunosuppressants for at least two weeks prior to sampling. The nasal microbiome was analyzed using 16S rRNA gene amplicon sequencing, and levels of 33 inflammatory cytokines were determined in nasal mucosal fluids using the MSD platform. Patients suffering from N-ERD and CRSwNP showed significantly worse smell perception and significantly higher levels of type 2 associated cytokines IL-5, IL-9, Eotaxin and CCL17. Across all 4 patient groups, *Corynebacteria* and *Staphylococci* showed the highest relative abundances. Although no significant difference in alpha and beta diversity was observed between the control and the CRS groups, pairwise testing revealed a higher relative abundance of *Staphylococci* in the middle meatus in N-ERD patients as compared to CRSwNP (p<0.001), CRSsNP (p<0.01) and disease controls (p<0.05) and of *Lawsonella* in patients suffering from CRSwNP in middle meatus and anterior naris in comparison to CRSsNP (p<0.0001 for both locations) and disease controls (p<0.01 and p<0.0001). Furthermore, we observed a positive correlation of *Staphylococci* with IL-5 (Pearson r=0.548) and a negative correlation for *Corynebacteria* and Eotaxin-3 (r=-0.540). Thus, in patients refraining from oral and nasal corticosteroid therapy for at least two weeks known to alter microbiome composition, we did not observe differences in microbiome alpha or beta diversity between various CRS entities and disease controls. However, our data suggest a close association between increased bacterial colonization with *Staphylococci* and decreased colonization by *Corynebacteria* as well as increased type 2 inflammation.

## 1 Introduction

Chronic rhinosinusitis (CRS) is a widespread and burdensome disease with a prevalence of approximately 11% in Europe. Up to a third of these patients suffer additionally from nasal polyps (CRSwNP), resulting in an estimated prevalence of 1.9 to 4% of the general population (1–3). This heterogeneous and multifactorial condition is primarily characterized by nasal obstruction and rhinorrhea but also causes loss of smell and facial pain (4) and may additionally be associated with asthma (5). A more severe syndrome combining CRSwNP, asthma, and hypersensitivity to aspirin and other non-selective cyclooxygenase-1 inhibitors is known as non-steroidal anti-inflammatory drug (NSAID)-exacerbated respiratory disease (N-ERD) (6). One characteristic feature of this disease is the presence of nasal polyps that frequently relapse after surgery, causing this disease to be challenging to manage (7).

CRS with and without NP (CRSsNP) is a complex, endotypically heterogeneous disease involving inflammatory and infectious factors. The latter have been widely explored in the last decade due to the broad usage of high-throughput sequencing techniques. The microbiome of healthy nasal sinuses is dominated by the genera *Corynebacterium, Staphylococcus, Streptococcus, Haemophilus and Moraxella* (8–10). The composition of the nasal microbiome may be involved in regulating epithelial barrier function and mucus production, as well as in the modulation of immune responses. Therefore it may play a role in numerous processes contributing to CRS pathogenesis (11). However, if and to what extent the diversity of the microbiome is affected in CRS or whether colonization by pathobionts, such as *Staphylococcus aureus*, contributes to the pathogenesis of the disease remains a matter of debate (12, 13). Though many studies investigated the microbiome in CRSwNP, there is scarce knowledge on the microbiome composition in patients suffering from N-ERD, which shows a very pronounced type 2 immune response profile (14). To the best of our knowledge, only one study so far compared N-ERD patients with healthy subjects and observed a reduced bacterial diversity and a loss of *Corynebacteria* in N-ERD patients (15). However, no other phenotype of CRS was analyzed in this study. In general, patients in microbiome studies are usually continuing their standard therapy for CRS including topical corticosteroids. The fact that this therapy may significantly alter microbial composition and to which extent this may also affect comparability of CRS with healthy patients is often not taken into account (16, 17).

To address the question of microbial composition in the context of the inflammatory environment in patients suffering from CRSsNP, CRSwNP, and N-ERD, we collected nasal mucosal lining fluids from the middle meatus and microbial swabs from both the middle meatus and the anterior naris of study patients (n=20 per group) and compared them to non-diseased controls (referred to as disease controls=DC). Allergic sensitization was similarly frequent among all four groups. Both sampling sites are known to reflect the sinus microbiome well (10, 12, 18, 19). Importantly, all patients refrained from oral and topical corticosteroid use two weeks before sampling. Additionally, we analyzed a large panel of 33 cytokines, and the microbiome composition using 16S rRNA gene amplicon sequencing and investigated correlations between the microbiome, the inflammatory environment and clinical outcome.

## 2 Material and methods

### 2.1 Study population and clinical measurements

After approval from the ethics committee of the Medical University of Vienna (EK No. 2027/2019), subject recruitment and clinical study were conducted at the Department of Otorhinolaryngology of the Medical University of Vienna. Written informed consent was obtained from each patient before study inclusion according to the Declaration of Helsinki. A total of 80 patients were recruited (20 per group for CRSwNP, CRSsNP, N-ERD, and DC).

CRS was diagnosed based upon the EPOS 2020 criteria (4). Furthermore, the diagnosis of N-ERD was defined according to the European Academy of Allergy and Clinical Immunology (EAACI) position paper as the presence of nasal polyps and asthma, in addition to developing respiratory symptoms upon the ingestion of aspirin or NSAIDs (6). In detail, 11 patients underwent a positive oral challenge test with NSAID. The remaining 9 patients had in the past either suffered from an anaphylactic shock (n=2), attended an emergency unit (n=3) or had reportedly repeated respiratory and nasal symptoms specifically after ingestion of NSAID. Exclusion criteria for participation in the study included age <18, pregnancy (standard urine pregnancy test was performed at the study visit), the use of nasal or systemic corticosteroids or immunosuppressants two weeks prior to their visit, use of biologicals, the presence of cystic fibrosis, and severe anatomic variations of the nasal cavity not allowing access with the nasal swabs.

During their visit to the outpatient clinic and after signing the informed consent, a medical history with regard to allergies, asthma, and nasal diseases, including previous surgeries and therapy, was recorded using a questionnaire. To assess the disease-specific and general health-related quality of life (QoL) in our study cohort, we used the sino-nasal outcome test-22 (SNOT-22), the EuroQol (EQ)-5D-3L, the Patient Health Questionnaire (PHQ)-2, and the asthma control test (ACT). Furthermore, scoring of polyp size was performed during routine nasal endoscopy by an otorhinolaryngologist. The total polyp score (TPS) system was used to grade nasal polyp size by nasal endoscopy. Both sides of the nasal cavity were separately assessed and scored, resulting in scores of 0–4 (0 = no polyps, 1 = small polyps in the middle meatus not reaching below the inferior border of the middle turbinate, 2 = polyps reaching below the lower border of the middle turbinate, 3 = large polyps reaching the lower border of the inferior turbinate or polyps medial to the middle turbinate, 4 = large polyps causing complete obstruction of the inferior nasal cavity) (20). The sum of both nostril scores was considered as the TPS. Olfactory testing was performed using the Sniffin Sticks Threshold (T), Discrimination (D) and Identification (I) test (Burghart, Holm, Germany). The sum of the three subtests resulted in the TDI score (21). For allergy assessment, a standard skin prick test was performed. Additionally, allergen-specific IgE levels and total IgE were determined in serum using the multiplex ALEX2 microarray (Allergy Explorer, MacroArray Diagnostics GmbH, Vienna, Austria). Patients’ characteristics and clinical measurements are displayed in **Table 1** and **Table E1**.

**Table 1:** Demographics and clinical characteristics of the study population.

Characteristics	N-ERD	CRSwNP	CRSsNP	DC
No.	20	20	20	20
Age (y; mean ± std deviation)	46.8 ± 10.0	49.4 ± 11.9	33.1 ± 10.0	25.8 ± 3.8
Sex: female, n(%)	8 (40)	4 (20)	8 (40)	10 (50)
Asthma, n(%)	20 (100)	16 (80)	5 (25)	1 (5)
Respiratory allergy, n(%)	8 (40)	8 (40)	7 (35)	9 (45)
SNOT-22 total (median; range)	48; 19-103	41; 9-76	46; 11-84	0; 0-11
TPS (median; range)	5; 0-8	4; 0-8	0	0
TDI (median; range)	12.5; 0-34.8	14; 7-24.8	32; 13-38	32.5; 14.5-37.5

N-ERD, NSAID-exacerbated respiratory disease; CRSwNP, Chronic rhinosinusitis with nasal polyps; CRSsNP, Chronic rhinosinusitis without nasal polyps; DC, disease control; y, years; std, standard; SNOT-22, sinonasal-outcome test 22; TPS, total polyp score; TDI, threshold discrimination and identification score.

### 2.2 Nasal sampling and cytokine measurement

First, nasosorptions were used for the collection of nasal secretions from the inferior nasal turbinate (Nasosorption FX-I, Hunt Developments (UK) Limited, Midhurst, West Sussex, United Kingdom) (22). The devices were inserted into the nasal cavity under visualization and placed along the lateral wall of the inferior turbinate. The patient held the device in place by pressing onto the external aspects of the alar and nasal cartilage for one minute. Thereafter the device was removed, placed on ice and immediately transported to the laboratory. The collected nasal fluid was eluted using 300 μL of phosphate-buffered saline (PBS) containing 0.05% Tween 20 and 1% bovine serum albumin (BSA) per nasosorption. Samples were immediately frozen at -80°C until further analysis for cytokines.

Thereafter, swabs optimized for the collection of microbiome specimens were applied (CLASSIQSwabs, Copan Diagnostics Inc. Murietta, CA, USA) to the anterior naris and middle meatus of each nostril. In addition, air controls were performed on each sampling day. Samples were immediately frozen at -80°C for later analysis by 16S rRNA gene amplicon sequencing.

Mediator concentrations in nasal secretions were measured using electrochemiluminescence technology through the use of the mesoscale discovery platform (MSD, Rockville, MA, USA). The following cytokines were analyzed: Eotaxin, Eotaxin 3, G-CSF, GM-CSF, IFN-γ, IL-10, IL-12p40, IL-12p70, IL-13, IL-15, IL-16, IL-17A, IL-1α, IL-1RA, IL-1β, IL-2, IL-3, IL-4, IL-5, IL-6, IL-7, IL-8, IL-9, IL-21, IL-22, IL-17E/IL-25, IL-27, IL-33, CCL17, TNF-α, TNF-β, TSLP, and VEGF-A. Custom cytokine plate kits were purchased from MSD and analysed according to the manufacturers protocol: Briefly, first linker-antibody solutions were prepared according to the protocol and then used to coat the U-Plex plate (50 μl per well), plates were sealed and left shaking overnight at 2°C–8°C. To generate the standard curve, the calibrator was diluted 1/10 in the provided metabolic assay working solution, and 8 standards were subsequently created with 1 in 4 dilution steps. After standard preparation was complete, the coated plates were washed 3 times with 150 μl of PBS-T (PBS 0.05% Tween 20) in each well. To each well, 50µL sample (pooled from right and left nostril of individual patients) or standard were added, the plate was sealed and incubated with agitation for 2 h at room temperature. Thereafter, plates were washed 3 times with PBS-T, and 50 μl of the provided detection antibody solution were added to each well, followed by another incubation period of 1 h while shaking. The plate was again washed 3 times with PBS-T, and 150 μl of the kit supplied MSD GOLD read buffer was added to each well. The plates were then analyzed using the MESO SECTOR S 600 plate reader (MSD), and cytokine concentrations were calculated using the DISCOVERY WORKBENCH software (MSD). Missing concentration values resulting from readings below or above the detection limit were set to lower or upper detection limit (**Table E2**) of the cytokine respectively.

### 2.3 Statistical analysis of clinical parameters

Statistical analyses of the clinical data were done using GraphPad Prism 8.4.1 (GraphPad Software, Inc., La Jolla, San Diego, CA, USA) after consultation with the Center for Medical Statistics, Informatics and Intelligent Systems, Medical University of Vienna. To normalize cytokine values for subsequent analysis, variables were transformed by log transformation. To test for statistically significant differences with regards to clinical parameters (i.e. TPS, SNOT-22) and cytokine concentrations between the 4 different groups, i.e. DC, CRSsNP, CRSwNP and N-ERD, the Kruskal-Wallis test was applied due to not normally distributed values (For results of Kruskal-Wallis tests for all figures, please refer to **Table E3**). This was followed by the Dunn’s test for multiple comparison. To report TPS, SNOT-22 and results of the quality of life questionnaires, descriptive analyses were used and described by median and range values. Statistical significance was defined as a P value of less than 0.05 and is indicated in the respective figures.

### 2.4 Microbiome analysis

16S rRNA gene sequencing and raw data processing was performed at the Joint Microbiome Facility of the Medical University of Vienna and the University of Vienna (project ID JMF-2102-04) as previously described (23). Samples were sequenced in two batches (runs). Amplicon sequence variants (ASVs) were inferred using the DADA2 R package v1.20 (24) applying the recommended workflow (25). FASTQ reads 1 and 2 were trimmed at 230 nt and 210 nt with allowed expected errors of 4 and 6, respectively. ASV sequences were subsequently classified using SINA version 1.6.1 (26) and the SILVA database SSU Ref NR 99 release 138.1 (27) using default parameters.

The resulting ASVs were stringently filtered to retain only trusted results. We only kept ASVs that were detected in at least 20% of non-negative control samples in both amplicon sequencing runs where the data was generated in; other ASVs were considered batch-effect contaminants. We also removed ASV_ku3_j7x (Homo sapiens mitochondrion) and ASV_6em_2nf, ASV_kip_nj9, ASV_paz_uyl based on manual inspection, as these were likely swab/reagent contaminant due to also being found in more than 4 negative controls. After merging the data from left and right nostril samples, all samples that had <1000 reads, as well as all samples from MN062, MN072, MN073, MN074 and MN075 (as these patients had a very high yield in their corresponding sampling negative control), were omitted from downstream analyses.

Following filtering, downstream analyses were performed using R v4.2 and Bioconductor v3.15 packages TreeSummarizedExperiment v2.4 (28), mia v1.4 (https://github.com/microbiome/mia), vegan v2.6.2 (https://CRAN.R-project.org/package=vegan), phyloseq v1.40 (29), microbiome v1.18 (http://microbiome.github.io), microViz v0.9.1 (30), DESeq2 v1.34 (31), ALDEx2 v1.28 (32). Alpha diversity was calculated using vegan and mia on rarified data (1007 read pairs). Beta diversity was calculated by performing a PCA on centered log ratio-transformed data using microViz. The difference in per-group centroids was tested with a PERMANOVA on Aitchison distance using vegan and microViz (**Table E4**). For PERMANOVA analysis, samples were grouped by disease group, presence and absence of allergy or asthma. Interactive effects between disease group and patient age, duration of CRS and number of polypectomy surgeries were tested, too. Pairwise differential abundance testing was performed using DESeq2 with alpha=0.05 and otherwise default parameters after adding a pseudocount of 1 to the data. Correlation between amplicon sequence variant (ASV) centered log-ratio transformed counts and environmental variables was calculated using ALDEx2’s correlation test using Pearson correlation with FDR multiple testing correction. Data accessibility: Amplicons datasets generated under JMF Project ID JMF-2102-04 and used in this study are deposited under the BioProject ID PRJNA907202.

## 3 Results

### 3.1 Patient cohort and clinical characteristics

**Figure 1:**
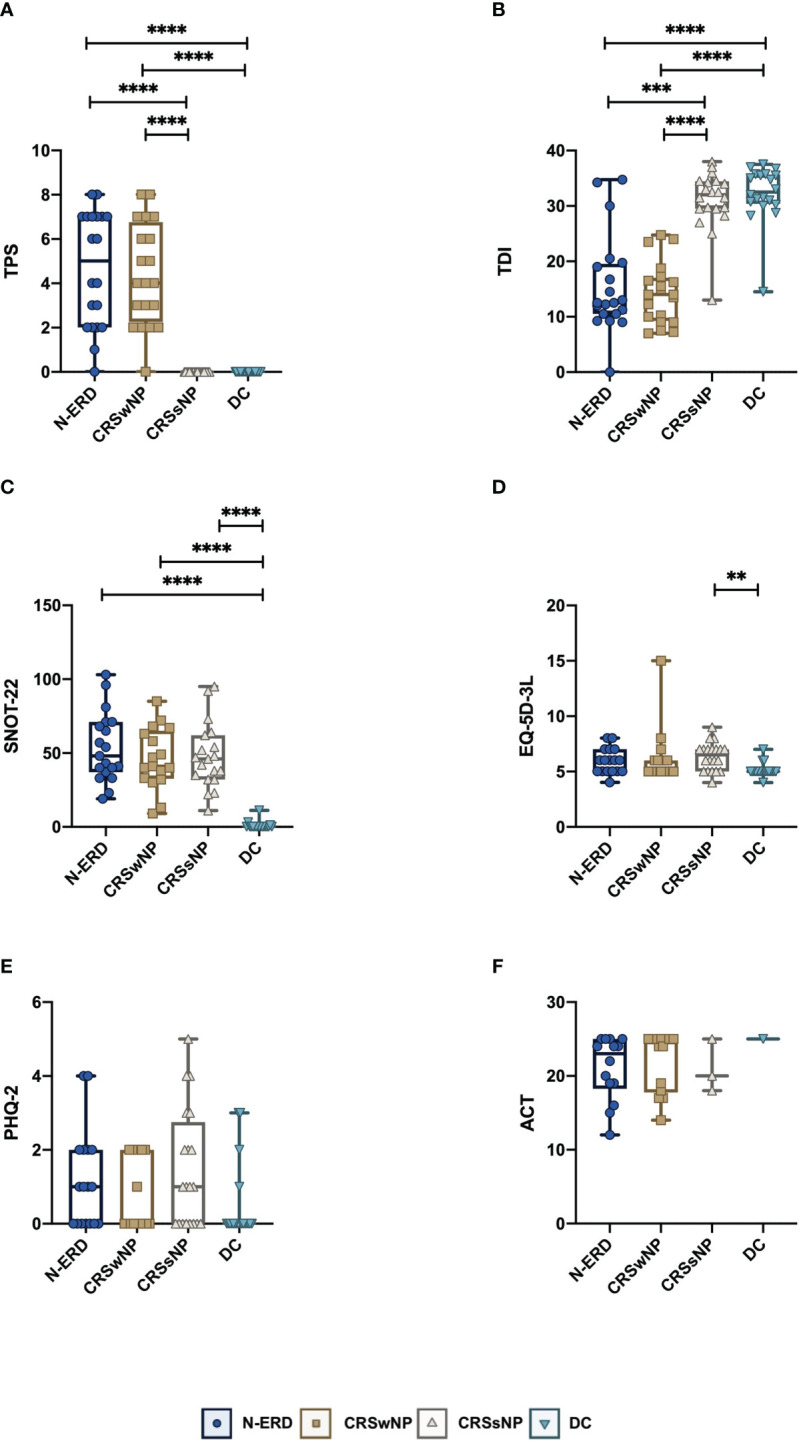
Clinical characteristics of patients suffering from chronic rhinosinusitis with (CRSwNP), without nasal polyposis (CRSsNP), non-steroidal anti-inflammatory drug-exacerbated respiratory disease (N-ERD) as compared to disease controls (DC). **(A–F)** Plots display values of **(A)** total polyp score (TPS), **(B)** threshold discrimination and identification score (TDI), **(C)** sinonasal-outcome test 22 (SNOT-22), **(D)** European Quality of Life 5 Dimensions 3 Level Version (EQ-5D-3L), **(E)** Patient Health Questionnaire-2 (PHQ-2) and **(F)** asthma control test (ACT) score in patients with CRSsNP (grey triangles, n=20 for all graphs except ACT: n=3), CRSwNP (brown squares, n=20 for all graphs except ACT: n=14) or N-ERD (dark blue circles, n=20 for all graphs except ACT: n=14) as compared to disease controls (DC, light blue triangles, n=20 for all graphs except ACT: n=1). Stars represent statistically significant differences between groups; using Kruskal-Wallis test (**Table E3**) followed by Dunn’s test (**P ≤ 0.01, ***P ≤ 0.001, ****P ≤ 0.0001). Line within each box represents the median, bottom border represents the 25^th^ percentile and top border the 75^th^ percentile of the data. Whiskers extend 1.5 times the interquartile range and diamond shaped points are outliers.

A total of 80 patients were included in this study (n=20 per group of DC, N-ERD, CRSwNP, CRSsNP). Patients suffering from N-ERD or CRSwNP had a mean age of 46.8 ± 10.0 and 49.4 ± 11.9 and were older than those suffering from CRSsNP (mean ± standard deviation 33.1 ± 10.0) or disease controls (mean ± standard deviation 25.8 ± 3.8) (**Table 1**, p-values: **Table E3** for Kruskal-Wallis and **Table E4** for PERMANOVA results). Comorbidities included respiratory allergies and asthma. The latter was more prevalent in patients with N-ERD (100%) and CRSwNP (80%). Allergic sensitization to respiratory allergens was similarly frequent among the 4 groups (Respiratory allergic subjects per group: DC n=9, CRSsNP n=7, CRSwNP n=8, N-ERD n=9). PERMANOVA analysis revealed that age, presence of allergy or asthma, number of previous surgeries as well as duration of CRS did not significantly influence microbiome composition (**Table E4**). TPS score tended to be higher in N-ERD (median 5; range 0-8) as compared to CRSwNP patients (median 4; range 0-8), but did not reach significance (**Figure 1A**). Both N-ERD (median 12.5; range 0-34.8) and CRSwNP (median 14; range 7-24.8) had significantly reduced smell perception as measured by TDI scores in comparison to CRSsNP (median 32; range 13-38) and disease controls (median 32.5; range 14.5-37.5), whilst no difference in TDI was observed between CRSsNP and DC (**Figure 1B**).

Regarding disease-specific and general health-related QoL, only the SNOT-22 showed significant differences between patients with CRS and DC (median 48; range 19-103 in N-ERD, 41; 9-76 in CRSwNP, 46; 11-84 in CRSsNP vs. 0; 0-11 in DC; P ≤ 0.0001) (**Figure 1C**). This difference was evident across all 4 subdomain analyses (nose, ear/face, sleep, emotion) with the nose domain yielding the highest scores (**Figure E1**). No major differences were observed in the EQ-5D-3L (**Figure 1D**), PHQ-2 (**Figure 1E**), and ACT questionnaires (**Figure 1F**). However, it needs to be noted that only one patient in the DC and three in the CRSsNP group suffered from asthma and completed the ACT. Therefore, the latter results need to be interpreted with caution.

### 3.2 Elevated levels of type 2 response-associated cytokines in nasal mucosal lining fluids of CRSwNP and N-ERD patients

**Figure 2:**
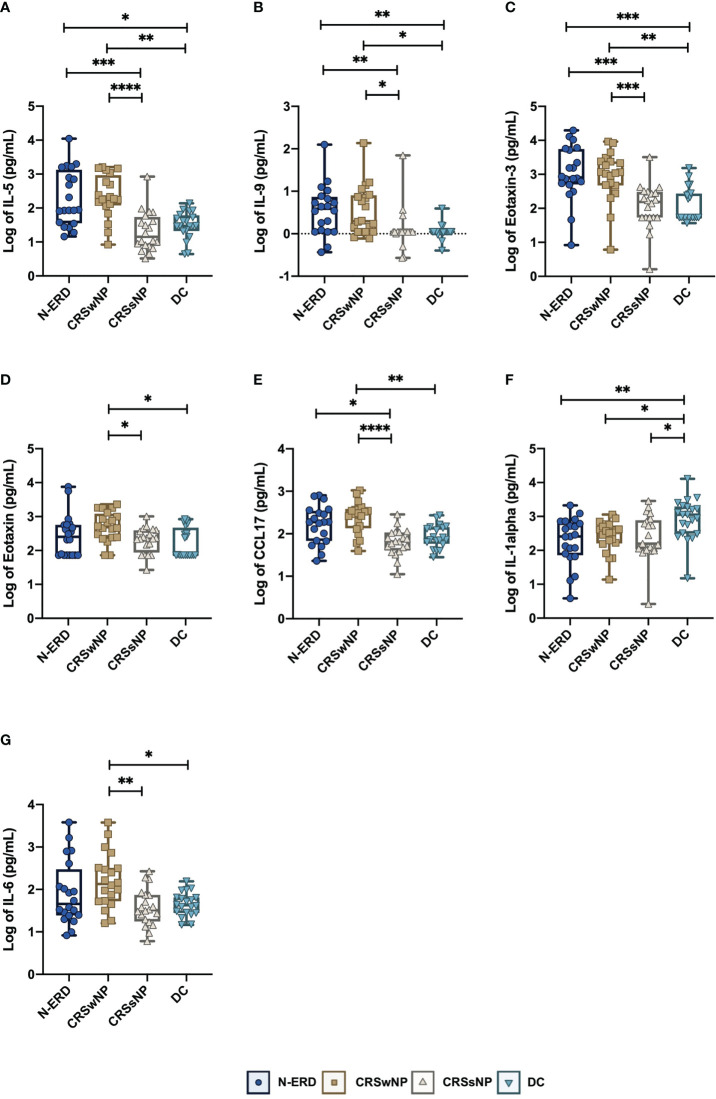
Selected mediator levels in nasal secretion in patients suffering from chronic rhinosinusitis with (CRSwNP), without nasal polyposis (CRSsNP), non-steroidal anti-inflammatory drug-exacerbated respiratory disease (N-ERD) as compared to disease controls (DC). (A-I) Levels of **(A)** IL-5, **(B)** IL-9, **(C)** Eotaxin-3, **(D)** Eotaxin, **(E)** CCL17, **(F)** IL-1α, **(G)** IL-6 are displayed as log-transformed mean concentration (y-axis, pg/ml) in patients with CRSsNP (grey triangles, n=20), CRSwNP (brown squares, n=20) or N-ERD (dark blue circles, n=20) as compared to disease controls (DC, light blue triangles, n=20). Stars represent statistically significant differences between groups using Kruskal-Wallis test (**Table E3**) followed by Dunn’s test (*P ≤ 0.05, **P ≤ 0.01, ***P ≤ 0.001, ****P ≤ 0.0001). Line within each box represents the median, bottom border represents the 25^th^ percentile and top border the 75^th^ percentile of the data. Whiskers extend 1.5 times the interquartile range and diamond shaped points are outliers.

Levels of a panel of 33 different cytokines were measured using the MSD Platform. We observed significantly elevated levels of the type 2 response-associated cytokines IL-5, IL-9 and Eotaxin-3 in the nasal secretions of patients with NP (N-ERD and CRSwNP) compared to patients without NP (CRSsNP and DC) (please refer to **Figures 2A–C** for p-values). Eotaxin was significantly elevated in CRSwNP patients only (**Figure 2D**), and CCL17 was significantly higher in CRSwNP and N-ERD patients than in CRSsNP and DC (**Figure 2E**). DC showed significantly higher levels of IL-1alpha than all other patient groups (**Figure 2F**). In contrast, the inflammatory cytokine IL-6 was mainly significantly elevated in CRSwNP patients (**Figure 2G**). All other cytokines did not show major differences between the different disease entities and disease controls (**Figure E2**).

### 3.3 Higher abundance of *Staphylococci* and lower levels of *Corynebacteria* in patients suffering from N-ERD as compared to the other patient groups

**Figure 3:**
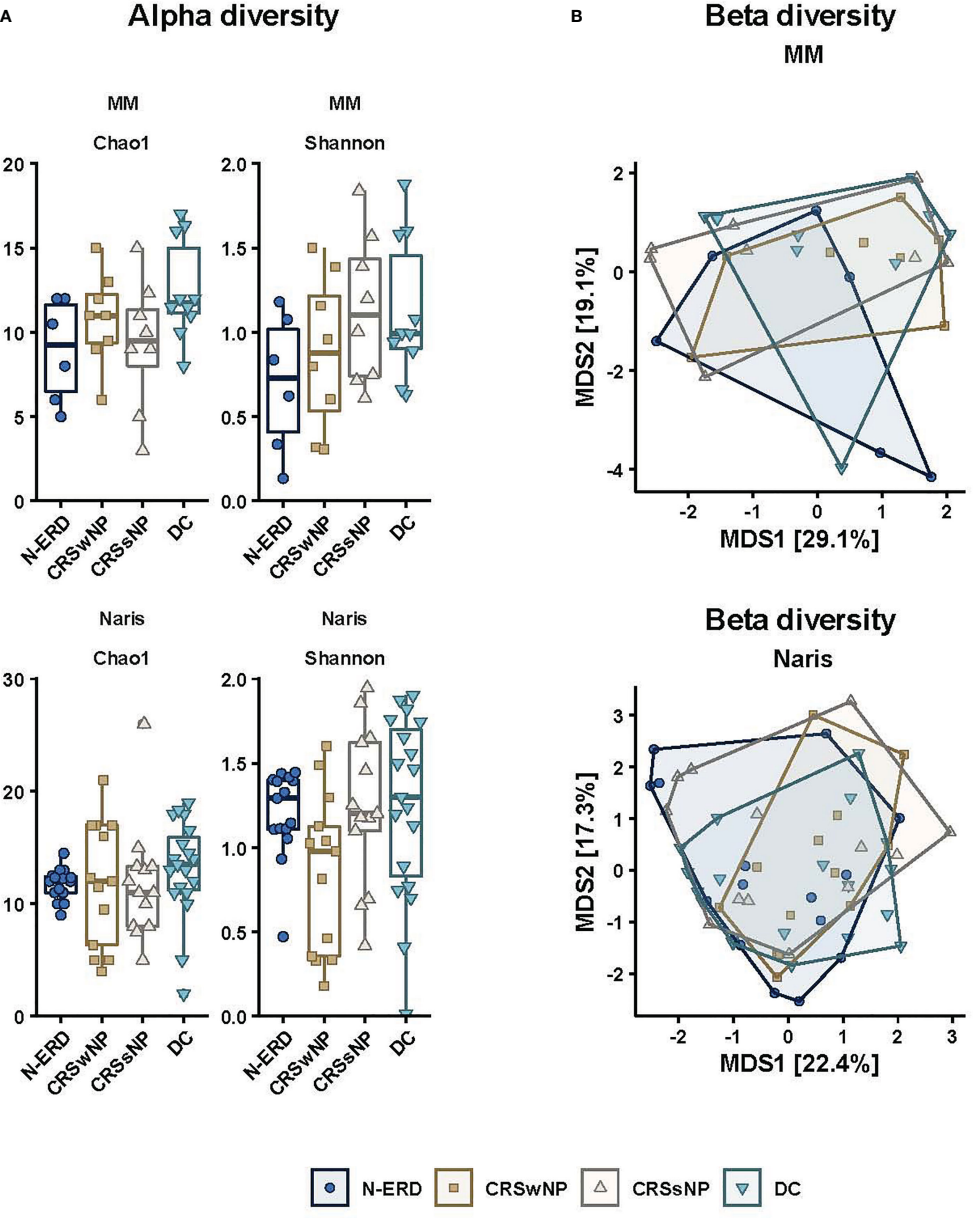
Alpha and beta diversity plots. **(A)** Alpha diversity divided into origin (anterior naris and middle meatus) and Chao1 richness values and Shannon diversity indices, **(B)** principal coordinates analysis plots (PCoA) showing beta diversity results of nasal microbial profiles divided into origin [anterior naris (AN) and middle meatus (MM)]; patients with CRSsNP (grey triangles, n_AN_=13, n_MM_= 8), CRSwNP (brown squares, n_AN_=13, n_MM_= 8) or N-ERD (dark blue circles, n_AN_=14, n_MM_= 7) as compared to disease controls (DC, light blue triangles, n_AN_=19, n_MM_= 11).

**Figure 4:**
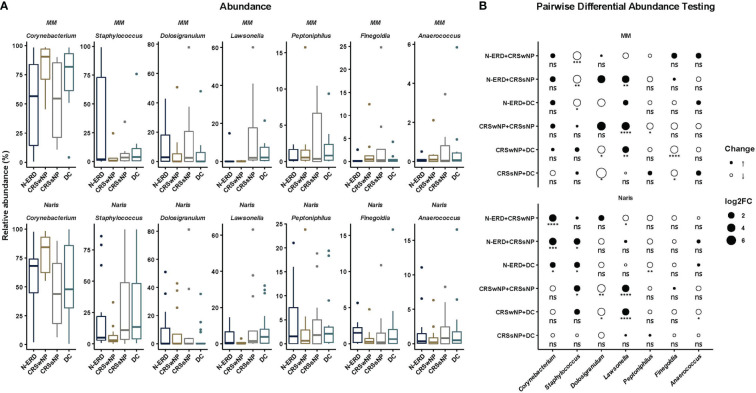
Microbiome taxonomic profiles by disease and origin in patients suffering from chronic rhinosinusitis with (CRSwNP) or without nasal polyposis (CRSsNP), or non-steroidal anti-inflammatory drug-exacerbated respiratory disease (N-ERD) as compared to disease controls (DC). **(A)** Relative abundance is displayed in percentage (y-axis) in patients with CRSsNP (grey; n_AN_=13, n_MM_= 8), CRSwNP (brown; n_AN_=13, n_MM_= 8) or N-ERD (dark blue; n_AN_=14, n_MM_= 7) as compared to disease controls (DC, light blue; n_AN_=19, n_MM_= 11). **(B)** Pairwise differential abundance testing was performed using DESeq2 with alpha=0.05. Log2 fold change (log2FC) is shown by size and color of circles [black = enlargement, white = reduction; circle size small = increase of abundance doubled, middle size = increase of abundance quadrupled, big size = increase of abundance sixfold; stars represent statistically significant differences between groups (*P ≤ 0.05, **P ≤ 0.01, ***P ≤ 0.001, ****P ≤ 0.0001, ns, non-significant)].

16S rRNA gene amplicon sequencing was performed in samples from the anterior naris (AN) and middle meatus (MM) in all patients. As low microbial biomass samples are more prone to be affected by exogenous contaminants which compromise data quality (12), the dataset was first stringently decontaminated, removing all ASVs detected as prevalent in PCR negative controls, DNA extraction blanks and air controls (see details in methods), and samples containing less than 1000 reads after decontamination were removed from the downstream analysis (available microbiome data: DC: n_AN_=19, n_MM_= 11; CRSsNP: n_AN_=13, n_MM_= 8; CRSwNP: n_AN_=13, n_MM_= 8; N-ERD: n_AN_=14, n_MM_=7). To assess intra-community diversity (alpha diversity), the Chao1 index focusing on total species richness, and the Shannon index assessing evenness in addition to richness, were calculated. For both indices, no significant differences between the patient groups and disease controls were noted (**Figure 3A**). To assess intersample variability, beta diversity was calculated and also showed no significant difference between the four groups, as depicted in multidimensional scaling plots in **Figure 3B**. ASVs affiliated with the genus *Corynebacteria* exhibited the highest relative abundance in both middle meatus and AN samples of all patients (**Figure 4A**). *Staphylococci, Peptoniphilus*, and *Finegoldia*-related ASVs showed a trend towards higher prevalence in AN of patients than in the MM. Lower relative abundance was observed for *Dolosigranulum* ASVs in AN samples. Pairwise differential abundance testing showed a significantly lower relative abundance of *Corynebacteria* in the AN of patients suffering from N-ERD and a higher relative abundance of *Staphylococci* in the MM of N-ERD patients as compared to other patients or disease controls (**Figure 4B**, p-values indicated in the figure). The genus *Dolosigranulum* was relatively less prevalent and the genus *Lawsonella* was relatively more prevalent in the nose of patients suffering from nasal polyps than in patients without polyps and disease controls. In the latter group, a higher relative abundance of *Finegoldia* was observed.

### 3.4 Correlation of *Staphylococci* and *Corynebacteria* with type 2 cytokines

**Figure 5:**
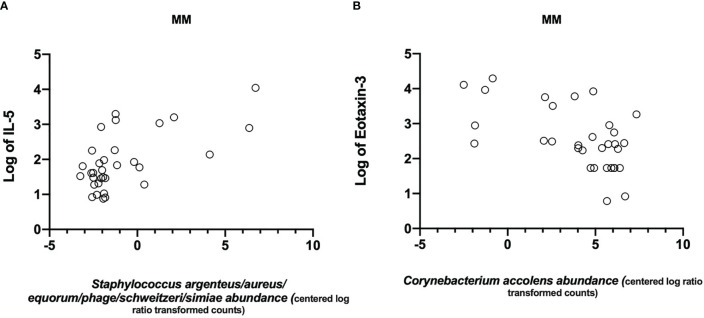
Significant correlations between mediator levels in nasal secretion and bacteria. Scatter plots showing correlations of **(A)** IL-5 (logtransformed) and *Staphylococcus argenteus*/*aureus*/*equorum*/*phage*/*schweitzeri*/*simiae* (Pearson r=0.55) and **(B)** a negative correlation between Eotaxin-3 (log-transformed) and *Corynebacterium accolens* (Pearson r=-0.54).

Last, we aimed to delineate associations between cytokine responses and clinical characteristics of patients with the relative abundance of different bacteria taxa. After adjustment for multiple testing, *Staphylococcus*-related ASV (*Staphylococcus argentus/aureus/equorum/phage/schweitzeri/simiae)* and IL-5 showed a moderate strong correlation (**Figure 5A**, Pearson r=0.55). At the same time, the relative abundance of an ASV classified as *Corynebacterium accolens* and Eotaxin-3 were negatively correlated in both MM and AN, but only showed a strong moderate correlation in MM (**Figure 5B**, Pearson r=-0.54).

## 4 Discussion

Here, we report on clinical characteristics, nasal cytokine levels and the nasal microbiome composition in disease controls, CRSsNP and CRSwNP, and, importantly, also N-ERD patients. As previously reported, N-ERD and CRSwNP had higher symptom burden and elevated levels of cytokines associated with type 2 responses than patients without polyps or disease controls. Though no significant difference in alpha and beta diversity was found in the nasal microbiome between the disease entities, pairwise differential abundance testing revealed that *Staphylococci* were relatively more prevalent in the middle meatus of N-ERD patients. Meanwhile, *Corynebacterium* was relatively less abundant in the anterior naris of these patients. Interestingly, specific ASVs related to *Staphylococci* were positively correlated with IL-5, thus strengthening the argument of the importance of the nasal microbiome-inflammatory cell axis in driving the disease.

Patients suffering from CRSwNP or N-ERD, and therefore suffering from polyps, showed severely impaired olfactory performance as assessed by the Sniffin’ Sticks test and diseases-specific quality of life as determined by SNOT-22. The associated significantly higher levels of IL-5 and Eotaxin-3 in nasal mucosal lining fluids are characteristic of the pronounced type 2 endotype in these patient groups (14, 33). Though a role for the nasal microbiome in olfactory function has been suggested (18, 34), we did not observe any association of the smell performance as assessed by TDI score with nasal microbiome composition. The sampling site may explain this different finding: While we took swabs from the middle meatus and anterior naris, studies mentioned above collected their samples directly at the nasal olfactory mucosa.

*Corynebacteria* and S*taphylococci* were the most abundant taxa in both the anterior naris and middle meatus of all patient groups. Commensals and opportunistic pathogens like *Staphyloccus, Dolosigranulum* or *Finegoldia* were slightly more prevalent in the anterior naris of our patients, which is in accordance with previous reports (18, 35). Nevertheless, our data confirm the suitability of both sampling sites as surrogates representing the sinus microbiome as they show a similar composition (36). Interestingly, some of the low abundant strains previously described as part of the nasal microbiome, like *Haemophilus* and *Moraxella* (8, 9), were not detectable in our samples. Potential explanations for this include the stringent filtering process in our analysis and environmental factors such as the different geographical locations or seasonal variations rendering our patients’ microbiome different from previous observations (9, 37).

There is conflicting evidence with regards to the diversity of microbiome between patients with or without chronic sinus inflammation: While some studies showed no statistical differences in indices determining microbiome diversity between diseased and non-disease patients (19, 38, 39), others argue for a lower diversity in those suffering from CRS (36, 40–43) or even distinct differences between CRSsNP and CRSwNP patients (44–46). Especially concerning the subtype N-ERD, knowledge about the nasal microbiome composition is scarce. To the best of our knowledge, only one study so far observed reduced diversity in N-ERD compared to disease controls, but no other forms of CRS were assessed in this study (15). Here, being the first to compare CRSsNP, CRSwNP and N-ERD patients with disease controls, we did not observe statistically significant differences in diversity between any of these groups using two complementary α diversity indices. It needs to be emphasized that we specifically asked patients included in this study to refrain from taking nasal or oral corticosteroids for at least two weeks prior to sampling. Due to their complex effect on epithelial cells, inflammatory cells and their mediators (47), corticosteroids have previously been reported to alter the nasal microbiome (16, 17): The use of corticosteroids may therefore account for some of the differences observed in other studies comparing CRS patients with corticosteroids as standard therapy to healthy controls.

Despite no overall difference in community diversity indices, using pairwise abundance comparison, we observed increased relative abundance of *Staphylococcus* in the middle meatus and a decreased relative abundance of *Corynebacterium* in the anterior naris of N-ERD patients as compared to other patient groups. A lower relative abundance of *Corynebacterium* has previously been observed in N-ERD patients in comparison to disease control subjects (15). It has also been associated with poor surgical outcomes (46). The associated increased levels of *Staphylococcus* in the middle meatus are in line with the notion of an interspecific competition between members of the two species (48–51). The observed positive correlation of a specific *Staphylococcus* ASV with the eosinophil attractant IL-5, while Corynebacterium *accolens* ASV negatively correlated with Eotaxin-3, further supports the recently put forward hypothesis that CRS is a continual spectrum of disease and that a shift in abundance of a limited number of specific microorganisms, such as *Staphylococcus aureus*, may be key to immune modulation and pathogenesis driving CRS. Simultaneously, other microorganisms, such as *Corynebacterium* species, more prevalent in the nose of healthy as compared to diseased patients, may have a protective function (13, 52). The association of IL-5 and a *Staphylococcus* related ASV (originating from either *S. aureus, S. argentus, S. equorum, S. phage, S. schweitzeri, or S. simiae)*, observed in this study may be a direct consequence of increased *Staphylococcus aureus* colonization as its derived products have been shown to stimulate mucosal tissue and underlying immune cells: Surface protein A can lead to mast cell degranulation whilst enterotoxins may induce Th2 cytokines amongst them IL-5 in cultured tissue fragments of nasal polyps (48). In this line, the presence of *S. aureus* enterotoxin specific IgE was associated with severe forms of CRSwNP characterized by strong eosinophilic inflammation, high total IgE concentration and asthma as comorbidity (53). This close link between microbiome composition and cytokine production is further supported by results from Wei et al. showing a higher prevalence of *Staphylococcus aureus* in the nasal mucosa of CRSwNP with high levels of serum eosinophils as compared to those with lower eosinophil numbers (39).

Pairwise differential abundance testing also revealed a trend towards *Lawsonella* being relatively more abundant in the nose of disease controls and CRSsNP, as compared to CRSwNP and partly N-ERD patients. *Lawsonella* species are considered part of the healthy nasal microbiome (51). Their decreased relative abundance might indicate sensitivity to inflammatory changes. In this context, *Lawsonella* was recently suggested as a biomarker for antibiotic usage as both CRSwNP and disease control patients actively taking antibiotics showed a significantly reduced relative abundance of *Lawsonella* (54).

Though we carefully selected our patients and ensured that they tested negative for COVID-19 as soon as tests were available at the time of recruitment, the study has some limitations. The groups were not equally balanced for age and sex, especially concerning the disease controls, which may have biased our results. However, as patients were recruited in a tertiary outpatient clinic during routine care, it was not possible to further stratify them by age. Patients in the disease control group were partly suffering from allergy, which may have influenced their microbiome. However, allergy was present to a similar percentage in patients from all four groups. Furthermore a PERMANOVA analysis was performed and confirmed that allergy was not a confounding factor, as no significant effect of allergy on microbiome composition was observed. We used a minimally invasive technique to collect nasal mucosal fluid, which had the advantage of collecting the cytokines directly at the nasal mucosa with a minimal dilution factor (55). This technique has been chosen as it causes no trauma as compared to cytokine detection in tissue homogenates collected by biopsies or during surgery (56). Nevertheless, cytokine concentrations in nasal fluids and tissue may differ and thus having not employed both techniques may be considered as a limitation of our study. Due to stringent controls and analysis algorithms, nasal microbiome samples of some patients could not be included in the analysis due to the low sequence yield/high degree of contaminants detected, but this is a well-known problem in the field. Lastly, the start of sample collection for our study coincides with the beginning of the COVID pandemic and thus most of the patients included wore facial masks at least during parts of the day (e.g., when shopping and using public transport) as a mandatory countermeasure of the Austrian government. This could also have impacted the nasal microbiome, but recent data has shown that masks affects the skin rather than the nasal microbiome (57).

In summary, we provide a comprehensive analysis of nasal inflammatory and microbial profiles of patients suffering not only from CRSsNP and CRSwNP but also N-ERD as compared to a control group without CRS. We observed a direct association of IL-5 levels with *Staphylococcus* colonization. Relative abundance of the latter was associated with decreased levels of *Corynebacteria* in the nose of the most severely diseased group, the N-ERD patients. Thus, we provide further evidence for the close link between the nasal microbiome and its inflammatory environment in CRS. Future studies are warranted to address the association between inflammatory markers and nasal microbiome in the steady state of the disease and acute exacerbations and comorbidities. Furthermore, the analysis of nasal microbiome from patients undergoing therapies with biologics targeting various components of the type 2 pathway may provide further insights into the role of community diversity and selected taxa in governing healthy and diseased state of the nasal mucosa.

## Data availability statement

The raw data supporting the conclusions of this article will be made available by the authors, without undue reservation.

## Ethics statement

The studies involving human participants were reviewed and approved by ethics committee of the Medical University of Vienna (EK No. 2027/2019). The patients/participants provided their written informed consent to participate in this study.

## Author contributions

TB, PP, BH, JED and SS designed the study. TB, NC, KF, DL, KG contributed to patient recruitment and sampling. TB, KG, VS, AT performed the experiments. TB, PP, BH and JED performed the data analysis. TB, BH, PP, JE-D and SS wrote the manuscript. All authors critically revised the manuscript. All authors contributed to the article and approved the submitted version.
