# Supplementary material for: The nasal microbiome in patients suffering from non-steroidal anti-inflammatory drugs-exacerbated respiratory disease in absence of corticosteroids
Source: Front Immunol. 2023 Apr 14;14:1112345. doi: 10.3389/fimmu.2023.1112345 (PMC10140405; doi:10.3389/fimmu.2023.1112345)
Supplement: Supplementary file 1 [file DataSheet_1.pdf]

Supplementary Material

The nasal microbiome in patients suffering from non-steroidal anti-inflammatory drugs-exacerbated respiratory disease (N-ERD) in absence of corticosteroids

Tina J. Bartosik[1], Nicholas J. Campion[1], Kilian Freisl[1], David T. Liu[1], Katharina Gangl[1], Victoria Stanek[1], Aldine Tu[1], Petra Pjevac[2,3], Bela Hausmann[2,4], Julia Eckl-Dorna[1*], Sven Schneider[1]

[1]Department of Otorhinolaryngology, General Hospital and Medical University of Vienna, Vienna, Austria
[2]Joint Microbiome Facility of the Medical University of Vienna and the University of Vienna, Vienna, Austria
[3]Department of Microbiology and Ecosystem Science, Centre for Microbiology and Environmental Systems Science, University of Vienna, Vienna, Austria
[4]Department of Laboratory Medicine, Medical University of Vienna, Vienna, Austria

*** Correspondence:**
Julia Eckl-Dorna, Department of Otorhinolaryngology, General Hospital and Medical University of Vienna, Vienna, Austria
julia.eckl-dorna@meduniwien.ac.at

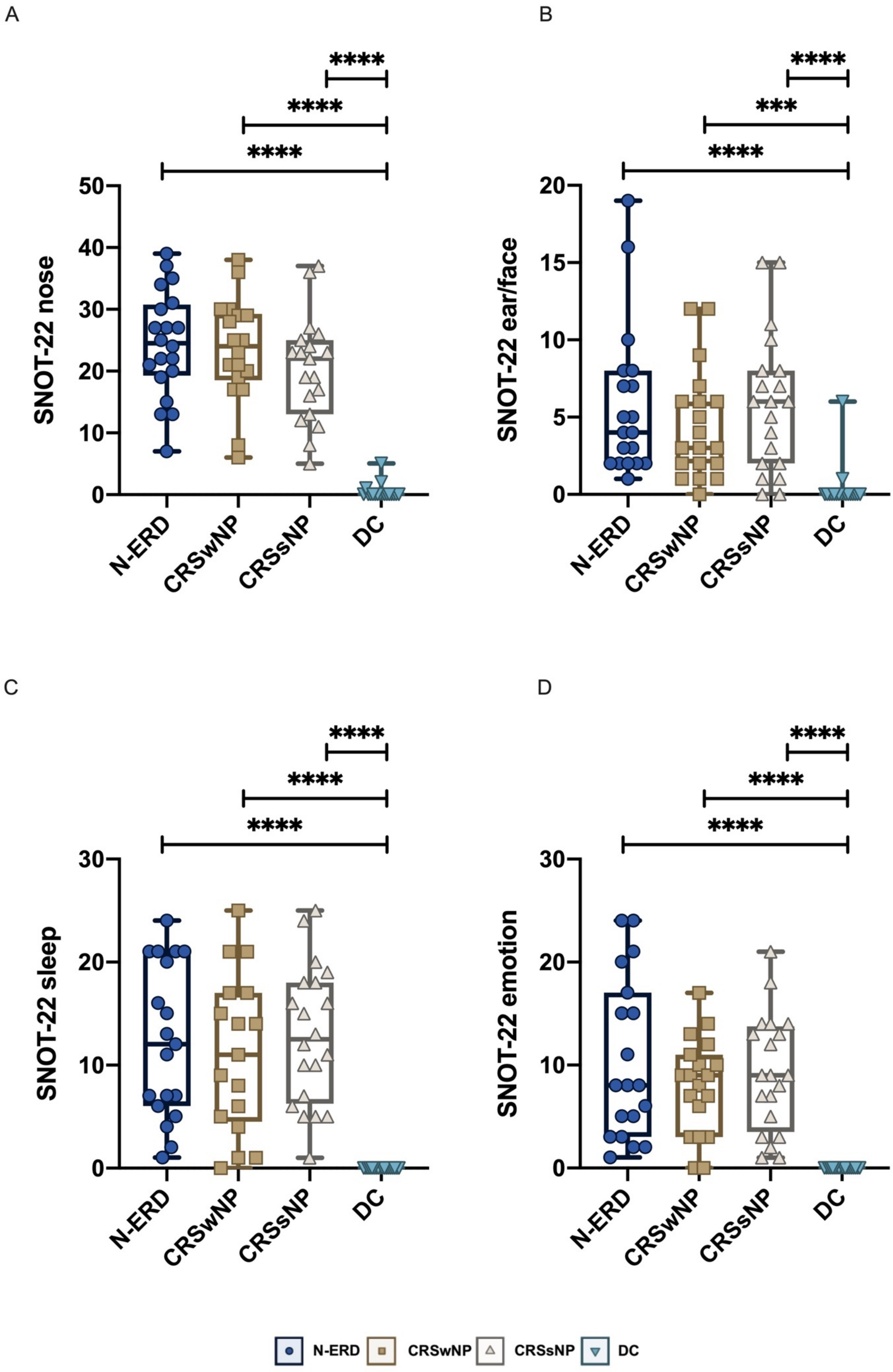

A
****
****
****
SNOT-22 nose
0
10
20
30
40
50
N-ERD
CRSwNP
CRSsNP
DC
B
****
***
****
SNOT-22 ear/face
0
5
10
15
20
N-ERD
CRSwNP
CRSsNP
DC
C
****
****
****
SNOT-22 sleep
0
10
20
30
N-ERD
CRSwNP
CRSsNP
DC
D
****
****
****
SNOT-22 emotion
0
10
20
30
N-ERD
CRSwNP
CRSsNP
DC
N-ERD
CRSwNP
CRSsNP
DC

Figure E1: Values of the sinonasal-outcome test 22 (SNOT-22) divided into subgroups
(A-D) Plots display values of (A) SNOT-22 nose (B) SNOT-22 ear/face (C) SNOT-22 sleep and (D) SNOT-22 emotion in patients with CRSsNP (grey triangles, n=20 for all graphs except ACT: n=3), CRSwNP (brown squares, n=20 for all except for ACT: n=14) or N-ERD (dark blue circles, n=20 for all graphs except ACT: n=14) as compared to disease controls (DC, light blue triangles, n=20 for all graphs except ACT: n=1). Stars represent statistically significant differences between groups using Kruskal Wallis test (Table E3) followed by Dunn's test (***: $P \leq 0.001$, ****$P \leq 0.0001$). Line within each box represents the median, bottom border represents the 25th percentile and top border the 75th percentile of the data. Whiskers extend 1.5 times the interquartile range and diamond shaped points are outliers.

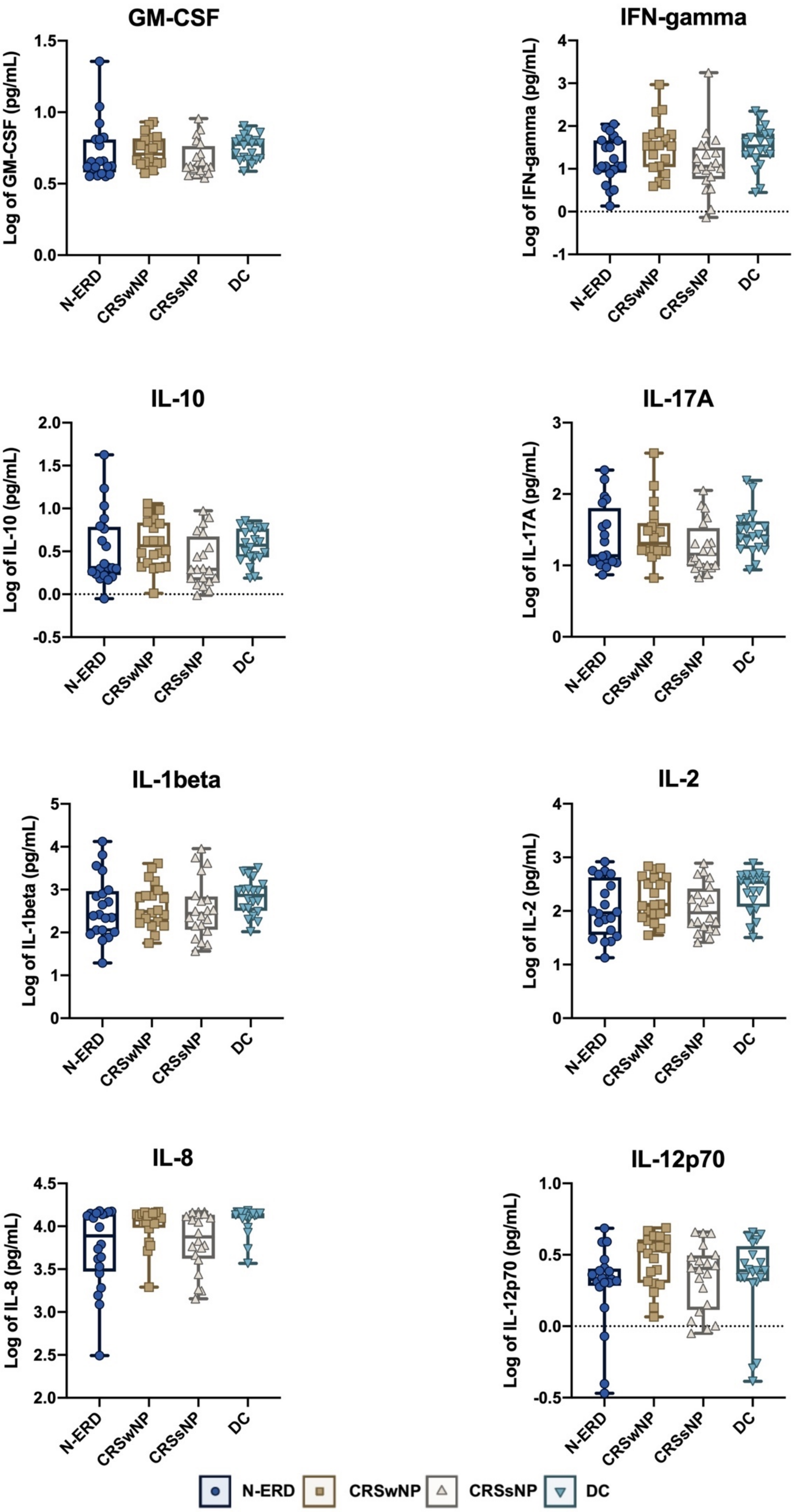
GM-CSF
Log of GM-CSF (pg/mL)
0.0
0.5
1.0
1.5
IFN-gamma
Log of IFN-gamma (pg/mL)
-1
0
1
2
3
4
IL-10
Log of IL-10 (pg/mL)
-0.5
0.0
0.5
1.0
1.5
2.0
IL-17A
Log of IL-17A (pg/mL)
0
1
2
3
IL-1beta
Log of IL-1beta (pg/mL)
0
1
2
3
4
5
IL-2
Log of IL-2 (pg/mL)
0
1
2
3
4
IL-8
Log of IL-8 (pg/mL)
2.0
2.5
3.0
3.5
4.0
4.5
IL-12p70
Log of IL-12p70 (pg/mL)
-0.5
0.0
0.5
1.0
N-ERD
CRSwNP
CRSsNP
DC

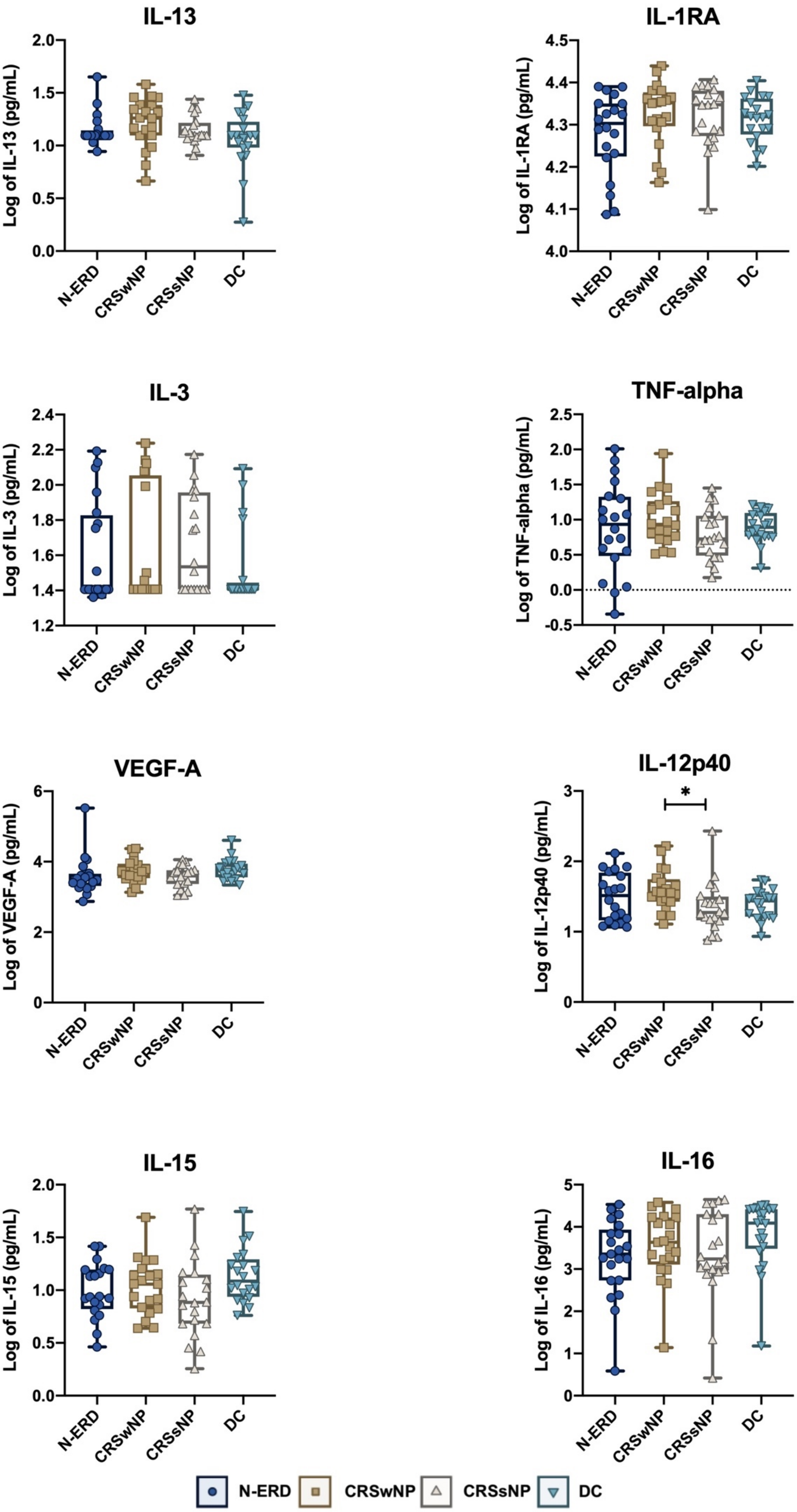
IL-13
Log of IL-13 (pg/mL)
0.0
0.5
1.0
1.5
2.0
IL-1RA
Log of IL-1RA (pg/mL)
4.0
4.1
4.2
4.3
4.4
4.5
IL-3
Log of IL-3 (pg/mL)
1.2
1.4
1.6
1.8
2.0
2.2
2.4
TNF-alpha
Log of TNF-alpha (pg/mL)
-0.5
0.0
0.5
1.0
1.5
2.0
2.5
VEGF-A
Log of VEGF-A (pg/mL)
0
2
4
6
IL-12p40
Log of IL-12p40 (pg/mL)
0
1
2
3
*
IL-15
Log of IL-15 (pg/mL)
0.0
0.5
1.0
1.5
2.0
IL-16
Log of IL-16 (pg/mL)
0
1
2
3
4
5
N-ERD
CRSwNP
CRSsNP
DC
N-ERD
CRSwNP
CRSsNP
DC

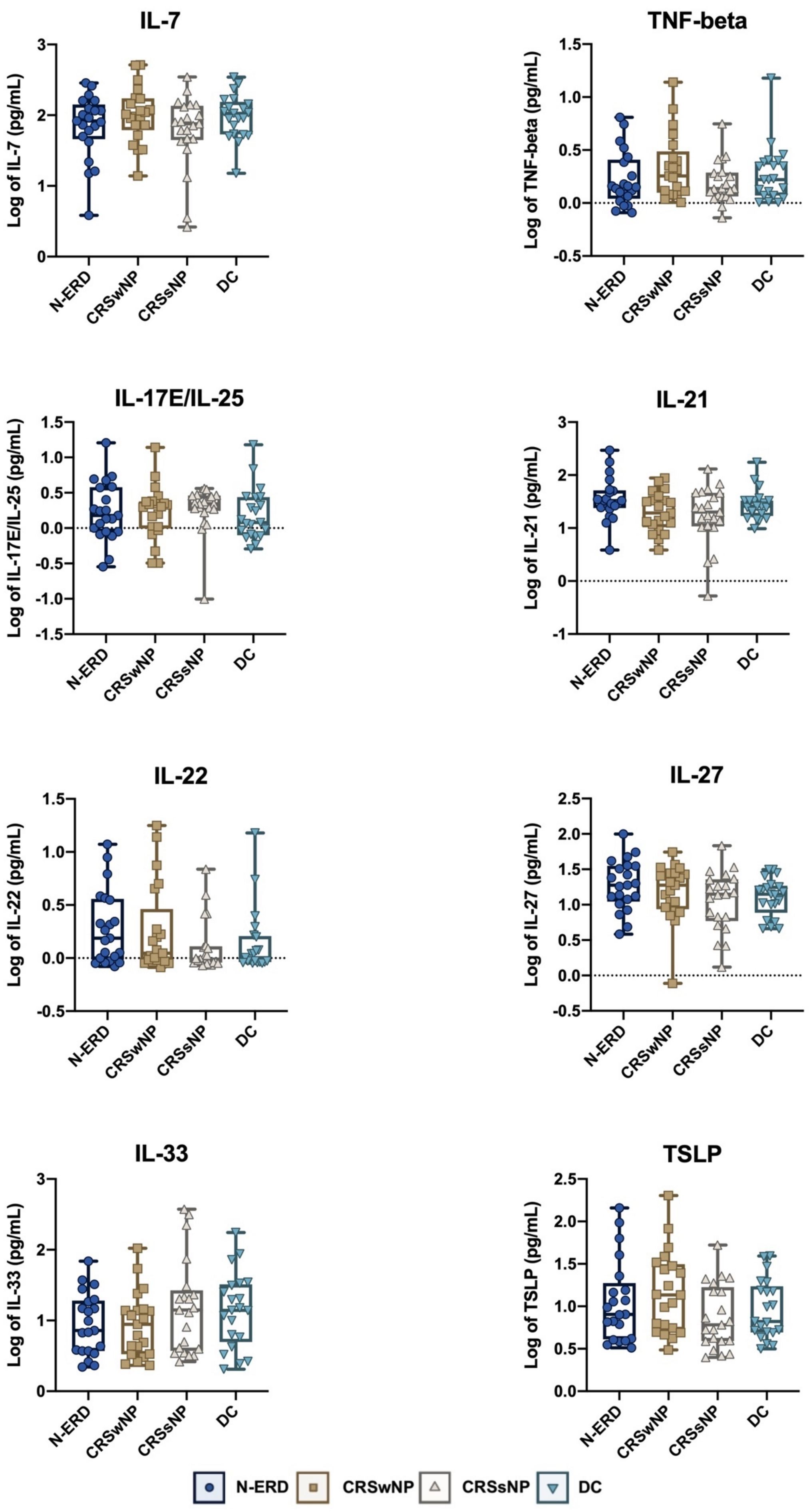
IL-7
Log of IL-7 (pg/mL)
TNF-beta
Log of TNF-beta (pg/mL)
IL-17E/IL-25
Log of IL-17E/IL-25 (pg/mL)
IL-21
Log of IL-21 (pg/mL)
IL-22
Log of IL-22 (pg/mL)
IL-27
Log of IL-27 (pg/mL)
IL-33
Log of IL-33 (pg/mL)
TSLP
Log of TSLP (pg/mL)
N-ERD
CRSwNP
CRSsNP
DC

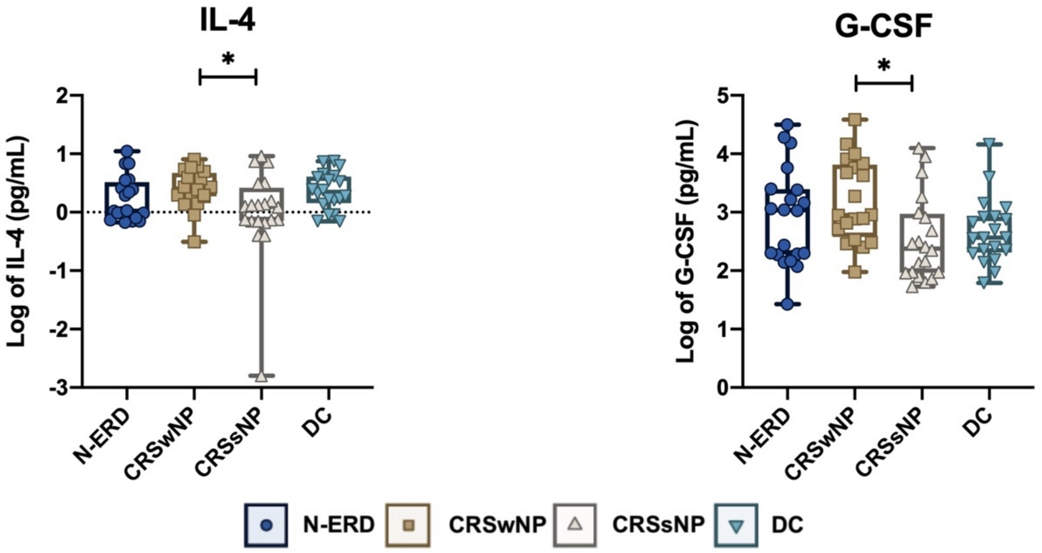

Figure E2: Selected mediator levels in nasal secretion in patients suffering from chronic rhinosinusitis with (CRSwNP), without nasal polyposis (CRSsNP), non-steroidal anti-inflammatory drug-exacerbated respiratory disease (N-ERD) as compared to disease controls (DC). Levels of G-CSF, GM-CSF, IFN-γ, IL-10, IL-12p40, IL-12p70, IL-13, IL-17A, IL-1RA, IL-1β, IL-2, IL-3, IL-4, IL-15, IL-16, IL-7, IL-8, IL-21, IL-22, IL-27, IL-33, IL-17E/IL-25, TNF-α, TNF-β, TSLP, and VEGF-A are displayed as log-transformed mean concentration (y-axis, pg/ml) in patients with CRSsNP (grey triangles, n=20), CRSwNP (brown squares, n=20) or N-ERD (dark blue circles, n=20) as compared to disease controls (DC, light blue triangles, n=20). Stars represent statistically significant differences between groups using Kruskal Wallis test (Table E3) followed by Dunn's test (*: $P \leq 0.05$). Line within each box represents the median, bottom border represents the 25th percentile and top border the 75th percentile of the data. Whiskers extend 1.5 times the interquartile range and diamond shaped points are outliers.

Patient number	Sex	Age (y)	Assigned group	Asthma	Respiratory allergy	Allergy according to skin prick test/ImmunoCAP	Total IgE (kU/L)	SNOT-22 total	TPS	TDI	MA AN	MA MM	Nr srg	CRS since (y)	Inhaled asthma treatment	N-ERD diagnosis
1	f	57	N-ERD	Y	N	none	230	71	2	12,25	N	N	3	33	Y	RNS
2	m	32	CRSwNP	Y	N	none	≤20	40	5	10	N	N	2	8	Y	
3	m	32	CRSwNP	Y	Y	HDM	≤20	nA	8	15	N	N	1	14	N	
4	f	49	CRSwNP	Y	Y	A, F, G, T	327	nA	2	23,5	N	N	2	32	Y	
5	f	54	N-ERD	Y	N	F	56	103	0	30	Y	N	3	13	Y	NC
6	m	42	N-ERD	Y	Y	HDM	≤20	96	8	10,5	N	N	2	12	N	NC
7	m	44	N-ERD	Y	N	none	71	65	7	9	N	Y	4	22	Y	NC
8	m	38	CRSwNP	Y	N	V	121	33	2	18,75	N	N	3	17	N	
9	m	64	N-ERD	Y	Y	A, F, G, T	77	41	7	20,5	N	N	1	37	Y	NC
10	m	57	N-ERD	Y	Y	A	≤20	48	2	11,25	Y	Y	1	18	Y	NC
11	m	63	CRSwNP	Y	Y	F, T	≤20	72	7	12,25	Y	Y	0	4	Y	
12	m	44	CRSwNP	N	N	V	≤20	41	5	10,25	Y	Y	2	27	N	
13	m	34	N-ERD	Y	Y	G, T	90	43	1	34,75	Y	N	1	4	N	NC
14	m	58	CRSwNP	Y	Y	F, G, T	67	47	4	24,75	Y	Y	1	10	Y	
15	f	22	DC	N	N	V	≤20	0	0	28,75	Y	N	0		N	
16	f	41	N-ERD	Y	Y	A, F, G, HDM, T	47	57	2	9,25	Y	N	1	6	N	NC
17	m	25	DC	N	N	F	≤20	0	0	36,75	Y	N	0		N	
18	m	24	DC	N	Y	F, HDM, V	≤20	11	0	35,5	Y	N	0		N	
19	f	29	N-ERD	Y	N	none	≤20	54	7	10,5	Y	Y	1	9	Y	EU
20	m	44	CRSwNP	Y	N	V	≤20	38	7	7,25	N	N	10	27	Y	
21	f	20	DC	N	N	none	≤20	3	0	34,75	Y	N	0		N	
22	m	58	CRSwNP	Y	N	none	≤20	49	4	17,25	Y	N	2	22	Y	
23	m	52	N-ERD	Y	Y	F, G	106	19	2	9,25	Y	N	4	22	Y	NC
24	m	49	CRSwNP	Y	Y	A, G, T, V	74	30	3	15,5	N	N	1	10	Y	

25	m	63	CRSwNP	Y	N	none	≤20	85	6	9	Y	N	3	15	Y	
26	m	43	CRSwNP	N	Y	A, G, HDM, V	80	40	0	9	Y	N	3	25	N	
27	f	29	DC	N	Y	A, F, HDM, G, T	170	0	0	31,5	Y	N	0		N	
28	m	39	CRSwNP	Y	N	V	56	9	2	24	N	N	1	12	Y	
29	m	25	DC	N	N	none	≤20	0	0	31	Y	Y	0		N	
30	m	26	DC	N	N	none	≤20	0	0	35,75	Y	Y	0		N	
31	f	21	DC	Y	Y	M	≤20	0	0	30,25	Y	Y	0		Y	
32	f	30	DC	N	N	none	≤20	0	0	35,5	Y	N	0		N	
33	m	35	DC	N	Y	F, G, T, V	126	0	0	30	Y	N	0		N	
34	m	50	N-ERD	Y	Y	T	≤20	23	3	12	Y	Y	2	22	Y	NC
35	f	41	CRSsNP	N	N	none	≤20	64	0	25	Y	N	2	12	N	
36	f	28	CRSsNP	N	Y	HDM, V	60	23	0	32,5	Y	N	0	12	N	
37	f	23	DC	N	Y	G	40	0	0	31,25	Y	Y	0		N	
38	f	21	DC	N	N	none	≤20	0	0	14,5	Y	Y	0		N	
39	f	25	DC	N	Y	F, HDM, T, V	115	nA	0	35,75	Y	Y	0		N	
40	m	30	DC	N	N	none	≤20	0	0	33	Y	Y	0		N	
41	m	25	CRSsNP	N	N	F	≤20	46	0	28,25	Y	Y	0	4	N	
42	f	35	CRSwNP	Y	N	none	≤20	32	7	7	Y	Y	1	12	Y	
43	m	25	DC	N	N	none	≤20	nA	0	28,25	Y	Y	0		N	
44	f	43	CRSwNP	Y	Y	A, G, HDM	80	67	3	16,5	Y	N	2	29	Y	
45	f	36	CRSsNP	N	N	none	≤20	92	0	29,5	Y	Y	3	7	N	
46	m	27	DC	N	N	none	≤20	0	0	35	Y	Y	0		N	
47	f	64	CRSwNP	Y	N	F, V	≤20	58	6	16,25	Y	N	5	22	Y	
48	m	26	DC	N	Y	A, F, G, T, V	74	0	0	32	N	N	0		N	
49	m	36	N-ERD	Y	N	none	≤20	37	6	16,75	Y	Y	0	4	N	EU
50	m	70	CRSwNP	Y	Y	M	236	68	2	7,5	Y	Y	1	13	Y	
51	m	67	CRSwNP	N	N	none	53	13	3	13,5	Y	Y	2	30	Y	

52	m	45	CRSwNP	Y	N	none	≤20	63	4	14	Y	Y	5	27	Y	
53	f	46	N-ERD	Y	Y	A, F, G, HDM, M, T	110	33	7	19	N	N	1	7	Y	AS
54	f	31	DC	N	N	V	≤20	1	0	37,5	Y	N	0		N	
55	f	46	N-ERD	Y	N	none	≤20	71	3	14,5	Y	N	0	5	Y	NC
56	f	40	N-ERD	Y	N	none	≤20	68	6	19,75	Y	N	2	22	Y	EU
57	m	63	N-ERD	Y	N	none	≤20	nA	7	nA	Y	N	6	21	Y	NC
58	f	29	DC	N	Y	A, F, G, HDM, T, V	397	0	0	30,75	Y	N	0		N	
59	m	54	CRSwNP	N	N	none	≤20	nA	8	14	Y	Y	2	19	N	
60	m	32	N-ERD	Y	N	none	≤20	40	4	34,25	Y	N	1	12	Y	RNS
61	f	51	N-ERD	Y	Y	F, G, HDM, M, V	38	40	7	12,5	Y	N	2	26	Y	RNS
62	f	44	CRSsNP	Y	N	none	≤20	95	0	13	Y	N	0	6	N	
63	m	46	N-ERD	Y	N	none	≤20	81	8	12,5	Y	Y	2	5	Y	AS
64	m	28	DC	N	Y	A, F, G, HDM	94	0	0	37	Y	Y	0		N	
65	f	48	CRSsNP	N	N	none	≤20	62	0	34,5	N	N	0	6	N	
66	m	54	N-ERD	Y	N	none	≤20	33	4	13	Y	N	2	9	Y	RNS
67	m	37	CRSsNP	N	N	none	≤20	38	0	31,5	Y	N	0	2	N	
68	m	37	CRSsNP	N	N	none	≤20	32	0	34,5	Y	Y	0	11	N	
69	f	22	CRSsNP	N	N	none	≤20	22	0	29,75	Y	Y	0	8	N	
70	m	26	CRSsNP	N	Y	G, T	≤20	32	0	34,25	Y	Y	0	2	N	
71	f	26	CRSsNP	N	N	none	≤20	35	0	33	N	N	0	8	N	
72	m	26	CRSsNP	N	Y	A, F	≤20	nA	0	27	N	Y	0	2	N	
73	m	52	CRSsNP	N	N	none	≤20	42	0	34	N	Y	0	3	N	
74	m	23	CRSsNP	Y	N	none	≤20	11	0	38	Y	Y	0	1	Y	
75	m	26	CRSsNP	N	Y	A	≤20	38	0	36	Y	Y	0	5	N	
76	m	42	CRSsNP	Y	N	none	≤20	47	0	37	Y	N	0	3	N	
77	m	32	CRSsNP	Y	Y	F, T, V	27	48	0	32,5	N	N	0	7	N	

78	m	23	CRSsNP	N	Y	A, G, HDM	646	73	0	29,75	Y	Y	0	3	N	
79	f	49	CRSsNP	N	N	none	≤20	54	0	31,5	Y	N	0	21	N	
80	m	20	CRSsNP	Y	Y	A, F, G, HDM, M, T, V	51	52	0	29,5	Y	Y	0	2	Y	

Table E1 Demographics of individual patients. Sex: M: Male, F: Female; Assigned group: DC: disease control; CRSwNP: Chronic rhinosinusitis with nasal polyps; CRSsNP: Chronic rhinosinusitis without nasal polyps; NERD: NSAID-exacerbated respiratory disease; Asthma: Y=Yes, N=No; Respiratory Allergy: Y=Yes, N=No; Allergy: A=Animal dander, F=Food, G= Grass, HDM= House dust mite; M=Mould, T=Tree, V=Venom; SNOT-22=sinonasal-outcome test 22; TPS=Total polyp score; MA=Microbiome analysis: Y=Yes, N=No; AN: anterior naris; MM=middle meatus; Nr srg= Number of surgeries (FESS, polypectomy); N-ERD diagnosis: NC=NSAID challenge, AS=anaphylactic shock, EU=emergency unit, RNS=repeated respiratory and nasal symptoms

**cytokines**	**Low detection limits**	**High detection limits**
GM-CSF	3.77	58500.00
IFN-γ	11.47	131500.00
IL-10	1.29	19800.00
IL-17A	8.57	122500.00
IL-1β	8.13	120650.00
IL-2	29.46	110100.00
IL-4	0.70	9600.00
IL-5	3.33	21850.00
IL-6	1.42	10150.00
IL-8	0.85	11450.00
Eotaxin	72.75	31500.00
Eotaxin-3	53.94	97000.00
IL-12p70	2.19	30650.00
IL-13	12.49	10600.00
IL-1RA	8.056	23300.00

IL-3	25.52	86000.00
IL-9	1.11	6850.00
TARC	21.65	14250.00
TNF-α	3.51	17450.00
VEGF-A	7.98	16850.00
G-CSF	7.87	96500.00
IL-12p40	8.378	115000.00
IL-15	1.79	15800.00
IL-16	10.02	99500.00
IL-1α	2.52	29450.00
IL-7	3.50	33250.00
TNF-β	1.81	20250.00
IL-17E/IL-25	3.65	35600.00
IL-21	32.91	131000.00
IL-22	0.99	14450.00
IL-27	18.94	144000.00
IL-33	3.35	46650.000
TSLP	3.20	40300.00

Table E2 Low and high detection limits of cytokines (pg/mL).

**variable**	**Kruskal-Wallis test (p-value)**
SNOT-22	<0.0001
SNOT-22 nose	<0.0001
SNOT-22 ear	<0.0001

SNOT-22 sleep	<0.0001
SNOT-22 emotion	<0.0001
TPS	<0.0001
TDI	<0.0001
ACT	0.6217
EQ-5D-3L	0.0054
PHQ-2	0.0702
G-CSF	0.0209
GM-CSF	0.0399
IFN-γ	0.0567
IL-10	0.0480
IL-12p40	0.0463
IL-12p70	0.1648
IL-13	0.0785
IL-17A	0.1070
IL-1RA	0.3752
IL-1β	0.2589
IL-2	0.1165
IL-3	0.3755
IL-4	0.0339
IL-15	0.1349
IL-16	0.1357
IL-7	0.3938
IL-8	0.0646
IL-21	0.1460
IL-22	0.2196

IL-27	0.2332
IL-33	0.4124
IL-17E/IL-25	0.3491
TNF-α	0.4009
TNF-β	0.4761
TSLP	0.2479
VEGF-A	0.0445
IL-5	<0.0001
IL-9	0.0002
Eotaxin-3	<0.0001
Eotaxin	0.0124
CCL17	<0.0001
IL-1α	0.0033
IL-6	0.0062

Table E3 Results of Kruskal-Wallis tests (p-value). Levels of IL-5, IL-9, Eotaxin-3, Eotaxin, CCL17, IL-1α , IL-6, G-CSF, GM-CSF, IFN-γ, IL-10, IL-12p40, IL-12p70, IL-13, IL-17A, IL-1RA, IL-1β, IL-2, IL-3, IL-4, IL-15, IL-16, IL-7, IL-8, IL-21, IL-22, IL-27, IL-33, IL-17E/IL-25, TNF-α, TNF-β, TSLP, and VEGF-A were log-transformed before testing.

A

**Factor**	**R2**	**PR (>F)**
Allergy	0.02911	0.505
CRS duration	0.67017	0.433
Number of surgeries	0.25987	0.197
Asthma	0.04725	0.144
Age	0.69046	0.144

B

Factor	R2	PR (>F)
Allergy	0.01676	0.431
CRS duration	0.49713	0.561
Number of surgeries	0.10603	0.776
Asthma	0.01768	0.394
Age	0.55242	0.357

Table E4 Permanova results of middle meatus (A) and anterior naris (B) Permutation test for adonis under reduced model Marginal effects of terms
